# Assessment of proliferating cell nuclear antigen (PCNA) expression at the invading front of oral squamous cell carcinoma

**DOI:** 10.1186/s12903-019-0928-9

**Published:** 2019-10-31

**Authors:** Emily Ming-Chieh Lu, Jithendra Ratnayake, Alison Mary Rich

**Affiliations:** Faculty of Dentistry, The University of Otago, PO Box 647, 310 Great King Street, Dunedin, 9016 New Zealand

**Keywords:** Proliferating cell nuclear antigen, Oral squamous cell carcinoma, Invasive tumour front, Cell proliferation, Prognosis

## Abstract

**Background:**

Accurate prediction of the behaviour of oral squamous cell carcinoma (OSCC) is necessary to determine prognosis and provide appropriate treatment. Therefore, it is important to investigate potential prognostic markers to determine their predictive ability. Histological assessment of specific features at the invading front of oral squamous cell carcinomas has shown to provide accurate and reproducible prognostic information. Proliferating cell nuclear antigen (PCNA) is a nuclear marker known to reflect cell turnover and may be used as a marker for tumour aggressiveness.

**Methods:**

Twenty cases of OSCC were histologically assessed to evaluate the correlation between proliferating cell nuclear antigen expression and invasive front grading. Each case was first assessed on a haematoxylin and eosin stained slide and an invading front grading (IFG) score was determined. In order to obtain a PCNA score, immunohistological staining was carried out using the peroxidase-labelled streptavidin-biotin technique with the monoclonal antibody PC10.

**Results:**

In all cases, tumour islands had a periphery of intensely stained proliferating cell nuclear antigen-positive epithelial cells. The average IFG score was 8 ± 1.8, and the average PCNA score was 75% ± 11.2. Regression analysis was done using data from the IFG score and PCNA score and taking the latter as the predictor variable. The Pearson correlation coefficient was 0.134, with a *p*-value of 0.572.

**Conclusion:**

Since the correlation between PCNA score and IFG score was not significant (*p* > 0.05), we conclude that there is no association between cell proliferation at the invading tumour front and the histological grading of OSCC.

## Background

Oral cancer is a serious and growing problem in many parts of the globe. Oral and pharyngeal cancer, grouped, is the sixth most common cancer in the world [1]. According to the global cancer statistics (GLOBOCAN 2018), 177,384 deaths were reported due to cancers of the lip and oral cavity, and it is common in high-risk regions such as South Asia [2, 3]. Despite advances in surgery and various adjunctive therapies, there is no evidence to suggest that the mortality of OSCC is increasing, and those that survive have to cope with debilitating side effects of treatment [4, 5].

Accurate staging is essential to evaluate treatment protocols and provide prognostic information for patients with oral squamous cell carcinoma (OSCC). Conventionally, this is based on the clinical assessment of tumour size, lymph node involvement and presence of distant metastases, the TNM system. The three parameters are tallied to give an overall stage; the higher the stage the worse the prognosis. While widely used, the TNM system has been criticised for its inability to predict survival in OSCC [6]. Modifications including the addition of the site of the tumour and a histopathological assessment led to the development of the STNMP system with a weighted numerical score for all five components [7]. While receiving some support, Langdon et al. found that the STNMP system was no more accurate in predicting survival than the TNM system [8] and STNMP staging is not in common usage [9].

Altered rates of cell proliferation are one of the hallmarks of tumour progression, and therefore, assessment of this feature may be useful in predicting patient prognosis [10, 11]. Proliferating Cell Nuclear Antigen (PCNA) is a nuclear protein and marker of cell proliferation. PCNA is strongly related to prognosis and survival in most types of solid malignancies, such as colorectal cancer and breast cancer [12–14]. It is known to accurately reflect rates of cellular proliferation and DNA synthesis since it accumulates in late G1 and early S phase [15, 16]. A dysregulation in cell proliferation could be assessed using PCNA, with an increase in PCNA immunoreactivity associated with an increase in cell proliferation [17].

Previous studies have demonstrated a positive correlation between the expression of PCNA and histological grading of OSCC. A difference in the expression of PCNA was found between normal and dysplastic epithelium [18] and between normal and malignant lesions [19]. Furthermore, PCNA expression showed a positive correlation with histological grading [20], with an increase in PCNA expression being associated with a poorly differentiated tumour and a reduced expression of PCNA suggestive of well-differentiated OSCC [21].

All of the aforementioned studies have, however, used the conventional histological grading system, which assesses the differentiation of the tumour as a whole. An alternative histological assessment is the invasive front grading (IFG) system which assesses the histological grading of the tumour at the invasive front only. The invasive tumour front (ITF) is a region of the tumour which contains cells with higher proliferative activity and [22] and a lower degree of differentiation [23]. Therefore, the ITF is considered the most important region of the tumour in determining prognosis and hence it was proposed that the IFG system would allow more accurate diagnosis and staging of cancer compared to the conventional histological grading [10].

The IFG system comprises four morphologic features; degree of keratinisation, nuclear polymorphism, pattern of invasion and host response [10], with each feature assigned a numerical value, generating an overall IFG score. The minimum score of 4 is the lowest IFG score obtainable and suggests a tumour with the best prognosis. Conversely, a maximum score of 16 suggests a tumour with the worst possible prognosis. So far, no studies have investigated the correlation between the expression of PCNA and IFG score at the ITF of OSCCs. Therefore, the main aim of this study was to investigate the correlation between the expression of PCNA at the invasive tumour front and the IFG histological grading in OSCCs.

## Methods

### Selection of study specimens

Ethical approval was obtained from the Faculty of Dentistry, University of Otago Human Ethics committee. Specimens used for the study were all formalin-fixed, paraffin-embedded tissues that had been diagnosed with OSCC from the Oral Pathology Centre, Faculty of Dentistry, University of Otago. For this study, oral squamous cell carcinoma (OSCC) arising in the oral cavity and lip vermilion was used. Specimens from the skin of the lips and face were excluded. The most recent cases of OSCC were reviewed until 20 suitable cases had been collected for the study. Inclusion criteria were the presence of the ITF, intra-oral site, patient consent to the use of the biological material after diagnostic procedures were complete for teaching and/or research purposes and there being sufficient material in the block. For each chosen case, a haematoxylin and eosin (H&E) slide was histologically assessed, and a PCNA score was obtained after immunohistological staining.

### Histological evaluation

Each H&E stained slide was assessed histologically according to the criteria outlined by Bryne [10] (Table 1). An IFG score was thus generated, where an increase in score is associated with a decrease in prognosis. Each H &E slide was initially evaluated by the primary author (EL) and later confirmed by AR, a consultant oral pathologist; this was repeated 2 weeks later to check the correlation. Where there was a discrepancy between examiners, the score from the most experienced examiner (AR) was taken. Each histological sample was then assigned an IFG score, as well as a conventional histological WHO grading [24].

**Table 1:** The Invading tumour front grading system used to generate total IFG score, as developed by Bryne et al. [10]

Morphological feature	Score			
	1	2	3	4
Degree of keratinisation	Highly keratinised (> 50% of the cells)	Moderately keratinised (20–50% of the cells)	Minimal keratinisation (5–20% of the cells)	No keratinisation (0–5% of the cells)
Nuclear polymorphism	Little nuclear polymorphism (> 75% mature cells)	Moderately abundant nuclear polymorphism (50–75% mature cells)	Abundant nuclear polymorphism (25–50% mature cells)	Extreme nuclear polymorphism (0–25% mature cells)
Pattern of invasion	Pushing, well delineated infiltrating borders	Infiltrating, solid cords, bands and /or strands	Small groups of cords of infiltrating cells (*n* > 15)	Marked and widespread cellular dissociation in small groups and /or in single cells (*n* < 15)
Host response (inflammatory cell infiltration)	Marked	Moderate	Slight	None

### Immunohistochemistry

A peroxidase-labelled streptavidin-biotin technique was performed, similar to previously described [20, 25]. Briefly, 4 μm thick tissue sections were deparaffinsed in xylene, rehydrated in graded absolute alcohols before washing in running water. The pre-treatment step involved treating the slides with proteinase K (DakoCytomation, Lot 00004344) for 10 min, followed by immersion in citrate buffer and heating in a microwave at 90 °C for 10 min. The slides were then allowed to cool to room temperature before being washed under running water. All slides were then treated with 3% H_2_O_2_ (BDH Laboratory Supplies Poole, England, Lot K32950980) in methanol for 15 min to block the endogenous peroxidase activity. To block nonspecific binding sites, 5% fetal calf serum (25ul frozen aliquot FCS: 5 ml PBS) was added. This was followed by application of the monoclonal anti-PCNA antibody PC10 (DakoCytomation, Lot 00005087). Sections were then treated with the biotinylated link and subsequently with the streptavidin horseradish peroxidase (LSAB kit, DakoCytomation, Lot 00003534). In order to visualise the peroxidase activity, slides were saturated with the chromogen, diaminobenzidine hydrochloride (DAB, DakoCytomation, Lot 016067) in a ratio of 1 ml DAB buffer: 1 drop DAB substrate. All of the previous steps were followed by a wash in PBS, unless otherwise stated. Finally, all slides were counterstained with Gills haematoxylin, washed in Scotts tap water before coverslips were applied.

All slides contained a section of normal epithelium to serve as a both positive and an internal control. A negative control in which the primary antibody (PC10) was omitted was included in each batch of slides stained.

### Immunohistochemical evaluation: expression of PCNA

Each specimen was orientated by firstly identifying the epithelium and then locating the tumour front in the deepest invading area of the tumour. Counting was done systematically, whereby malignant keratinocytes in alternating high power fields at the invading front were counted until a total of 200 cells was reached. A PCNA positive cell was defined as one with clear, distinct brown nuclear staining. The number of positive cells was expressed as a percentage of the total number counted to generate a PCNA score [25]. Intensity of PCNA expression was recorded with reference to a known positive control and was reviewed and verified by a specialist oral pathologist. Grade 1 denoted intense PCNA staining, grade 2 moderate PCNA staining and grade 3 indicated weak staining. The calibration process was carried out on a total of six slides where agreement was reached for each of the three grading intensities. Following calibration, each slide was assessed independently by two examiners. The final number of PCNA positive cells recorded for each slide was obtained from taking the average positive cell counts from the two examiners, EL and AR. An appropriate level of inter-examiner agreement was achieved across all slides (Cohen’s Kappa coefficient > 0.6). To aid in the comparison between slides, images of the cells in the invading front were captured digitally for later reference.

### Statistical analysis

Regression analysis was performed using Statistical Package for Social Studies software (SPSS version 24; IBM Corporation, Armonk, NY, USA) to compare variables. The level of significance was set at *p* < 0.05).

## Results

### Invasive front grading

**Fig. 1:**
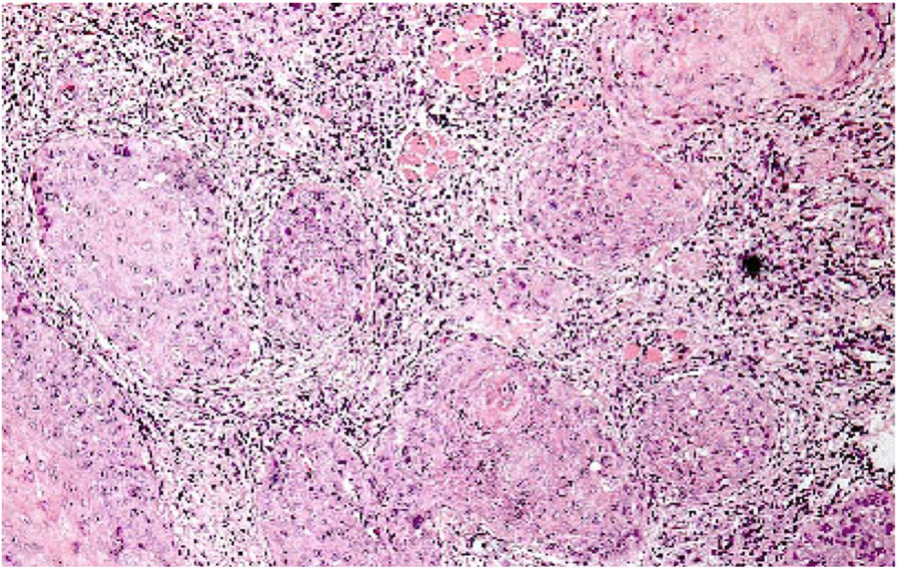
H& E section showing malignant epithelial islands at the ITF (×10 objective lens). The total IFG score was 7 (degree of keratinisation = 1; nuclear polymorphism = 3; pattern of invasion = 2; host response = 1)

At the invading front of each specimen, four morphological features were assessed on H&E slides as outlined in Table 1. Each H&E section was assessed histologically for their IFG scores according to the criteria outlined by Bryne [10], and matched with their existing conventional histological WHO grading [24]. The total IFG score and the scores for each morphological feature for each specimen are shown in Table 2. The average IFG score was 8 with a standard deviation (SD) of 1.84. The mode score was 7, and the score range was between 5 and 13 inclusive. The mode score for each morphologic feature was also calculated (Table 3). A mode score of 1 for “host response” indicated a marked host response in most of the specimens. Both “Degree of keratinisation” and “pattern of response” had a mode score of 2, indicating that in most cases 20–50% of cells were keratinised cells at the tumour front, and there were infiltrating, solid cords, bands or strands invading the connective tissue. Lastly, “nuclear polymorphism” had a mode score of 3, indicating that 25–50% mature cells at the invading front showed this feature (Table 3). Figure 1 shows the H & E section of a specimen and its IFG score.

**Table 2:** H&E histological evaluation using the IFG grading system [10]

OSCC cases	Degree of keratinization	Nuclear polymorphism	Pattern of invasion	Host response	Total score
1)	1	3	2	1	7
2)	1	2	2	1	6
3)	2	1	1	1	5
4)	2	3	1	1	7
5)	2	4	1	2	9
6)	1	3	2	1	7
7)	2	3	2	2	9
8)	3	3	2	1	9
9)	2	3	3	1	9
10)	3	4	3	3	13
11)	2	3	2	3	10
12)	2	4	3	1	10
13)	2	3	2	1	8
14)	1	2	2	1	6
15)	1	2	3	1	7
16)	1	2	2	1	6
17)	1	4	2	2	9
18)	3	2	1	1	7
19)	2	2	2	2	8
20)	1	2	3	2	8

**Table 3:** Invasive front grading (IFG). Mode scores for individual morphologic feature

Morphologic features	Mode score
Degree of keratinisation	2
Nuclear polymorphism	3
Pattern of invasion	2
Host response	1

### PCNA quantification and expression

Table 4 shows the PCNA score (%), and the intensity of PCNA immunostaining expressed as a grade for each specimen. PCNA scores were computed by quantifying the number of cells with a distinct brown nuclear staining (PCNA positive cells) out of a total of 200 cells counted at the invading front. Intensity of staining was compared to a known control and defined as 1 = intense, 2 = moderate or 3 = weak. The average PCNA score was 75.0%, SD 11.2%. PCNA expression was intense (grade 1) in 45% of the cases, moderate (grade 2) in 40% of the cases, and weak (grade 3) in 15% of the cases. The mode intensity score was 1.

**Table 4:** Summary of PCNA scores (%), intensity of PCNA staining, IFG scores and conventional histological grading scores for OSCC cases

OSCC cases	No. of PCNA +ve cells	PCNA score % (out of 200)	Intensity of staining	IFG Score	Conventional histological grading
1)	184	92	2	7	1
2)	103	51.5	3	6	1
3)	157	78.5	2	5	1
4)	169	84.5	2	7	1
5)	127	63.5	1	9	2
6)	137	68.5	3	7	1
7)	176	88	1	9	2
8)	163	81.5	1	9	2
9)	139	69.5	1	9	1
10)	148	74	2	13	2
11)	179	89.5	2	10	2
12)	149	74.5	1	10	1
13)	120	60	2	8	1
14)	165	82.5	1	6	1
15)	139	69.5	1	7	1
16)	147	73.5	2	6	1
17)	150	75	1	9	1
18)	116	58.2	3	7	2
19)	178	89	1	8	1
20)	154	77	2	8	1

**Fig. 2:**
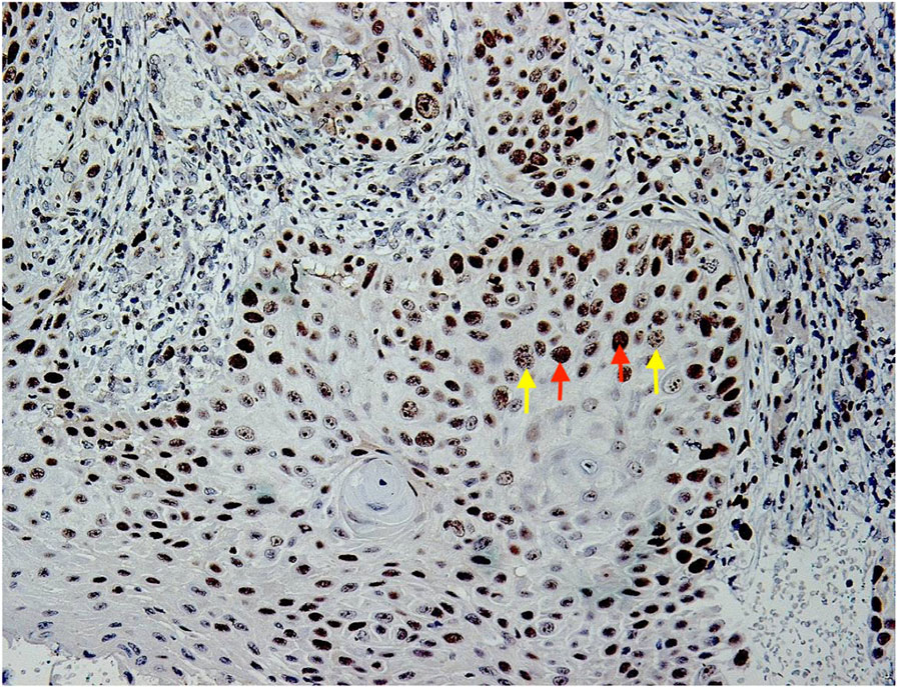
PCNA expression at the ITF showing intensely stained nucleus (red arrow), surrounded by granular prickle cells (yellow arrows). (×20 objective)

In all cases, epithelial islands at the tumour front had a periphery of intensely stained PCNA positive epithelial cells, surrounding prickle cells (Fig. 2). The prickle cells were in most cases moderately or weakly stained. However, no staining was observed in some specimens. Many islands had an inner core of keratin, which was unstained.

### Regression analyses

To assess the relationship between PCNA expression and IFG and taking the intensity of staining as the predictor variable, the following model was generated: IFG = 9.333–7.84 intensity. The *p*-value was 0.179, and hence there was no association between intensity of PCNA staining and IFG score in this study (Table 4). Using PCNA score as the predictor variable, no significant relationship was found between PCNA score and intensity of PCNA staining (*p* = 0.068) (Table 4). A significant relationship (*p* = 0.012) between IFG score and the conventional histological grading system was found with regression analysis using IFG score as the predictor variable (Table 4). To determine whether there was any relationship between PCNA score and IFG score, PCNA score was taken as the predictor variable (Table 4) and the regression line generated follows the model: IFG = 6.343 + 0.022PCNA. This association was not significant (*p* > 0.05). The Pearson correlation coefficient (R) was 0.134 with a *p*-value of 0.572. In addition, the Spearman correlation coefficient (ρ) was found to be 0.143; *p* = 0.547.

## Discussion

The present study sought to assess the correlation between the expression of PCNA at the ITF and the IFG score of OSCC cases from archival blocks. Our study showed that there was an average of 75% PCNA positive cells at the ITF. It is difficult to compare this finding with that in the literature as most studies investigating PCNA expression were exclusive to the ITF [17, 18, 26] and the one study [27] which specifically examined the expression of PCNA at the ITF did not stipulate the WHO grading and the location of OSCC samples in the oral cavity.

The intensity of PCNA positivity were mostly either intense or moderate (45 and 40% of cases respectively). This intense positivity observed at the periphery of the malignant epithelial islands has been previously noted [19, 26, 28] and suggests that this is the main site of cellular proliferation. The variation in PCNA expression, as observed in this study could be related to technical factors such as duration of fixation, size of tissue block and the type of fixative [29]. While the latter was consistent across all cases in this study, the first two factors could not be controlled. Furthermore, the variability in marker expression could be due to the fact that PCNA has a long half-life of 20 h [30]. Therefore, it is possible that non-cycling cells may show residual PCNA expression and result in an overestimation of the proliferating population.

The histological assessment using the IFG generated scores suggested that most cases in this study had a reasonable prognosis (Table 3). There was also positive association between IFG scores and the conventional histological grade, suggesting a consistency between the two systems [10].

Overall, the study showed no association between the expression of PCNA (percentage score and intensity grading) and the IFG score at the ITF. While the data seems to contrast with earlier studies which showed a positive correlation between PCNA and histological grade [21, 26], it is consistent with the findings from Tsai and Jin which showed a lack of correlation between PCNA expression and histological grading [17]. All of the aforementioned studies have used the conventional histological grading in its assessment, rather than the ITF grading system employed in this study [10].

The lack of correlation between PCNA expression and histological grading should not be unexpected, however, given that there is evidence to suggest an absence of an association between PCNA expression and patient survival [31, 32]. A more recent study also showed that the expression of proliferation markers Ki67 and AgNOR at the ITF were not associated with prognosis of OSCC [33]. However, overexpression of PCNA in conjunction with p53 was associated with OSCCs with a poor prognosis [27]. Similarly, overexpression of PCNA and EGFR was associated with poor survival [34]. This suggests that elevated levels of PCNA, in the presence of increased expression of a supplementary biomarker improves prognostic determination. Therefore, the long-term follow-up of our patient cases would determine whether the IFG grading system and/ or PCNA score has any relationship with patient survival.

## Conclusions

The study showed no correlation between the expression of PCNA at the ITF and the IFG scores of OSCCs. Therefore, there was no association between cell proliferation activity and histological grading at the invading tumour front.

## Data Availability

The datasets used and/or analysed during the current study are available from the corresponding author on reasonable request.

## Abbreviations

IFG: Invading front grading
ITF: Invasive tumour front
OSCC: Oral squamous cell carcinoma
PBS: Phosphate buffer saline
PCNA: Proliferating cell nuclear antigen
